# The role of Annexin A3 in coronary arterial lesions in children with Kawasaki disease

**DOI:** 10.3389/fped.2023.1111788

**Published:** 2023-02-14

**Authors:** Mengling Li, Dong Liu, Fengchuan Jing, Ruixi Liu, Qijian Yi

**Affiliations:** ^1^ Department of Cardiovascular Medicine, National Clinical Research Center for Child Health and Disorders, Ministry of Education Key Laboratory of Child Development and Disorders, China International Science and Technology Cooperation Base of Child Development and Critical Disorders, Children's Hospital of Chongqing Medical University, Chongqing, China; ^2^ Department of Pediatrics, Sichuan Mianyang 404 Hospital, Mianyang, China; ^3^ Chongqing Key Laboratory of Pediatrics, Chongqing, China

**Keywords:** Kawasaki disease (KD), coronary arterial lesions, Annexin A3 (ANXA3), platelet count (PLT), pathogenesis

## Abstract

Kawasaki disease (KD) is an acute, self-limited vasculitis, and the etiology is still unclear. Coronary arterial lesions (CALs) are a major complication of KD. Excessive inflammation and immunologic abnormities are involved in the pathogenesis of KD and CALs. Annexin A3 (ANXA3) plays crucial roles in cell migration and differentiation, inflammation, cardiovascular and membrane metabolic diseases. The purpose of this study was to investigate the effect of ANXA3 on the pathogenesis of KD and CALs. There were 109 children with KD in the KD group [which was divided into two groups: 67 patients with CALs in the KD-CAL group, and 42 patients with noncoronary arterial lesions (NCALs) in the KD-NCAL group] and 58 healthy children in the control (HC) group. Clinical and laboratory data were retrospectively collected from all patients with KD. The serum concentration of ANXA3 was measured by enzyme-linked immunosorbent assays (ELISAs). Serum ANXA3 levels were higher in the KD group than in the HC group (*P* < 0.05). There was a higher concentration of serum ANXA3 in the KD-CAL group than in the KD-NCAL group (*P* < 0.05). Neutrophil cell counts and serum ANXA3 levels were higher in the KD group than in the HC group (*P* < 0.05) and quickly decreased when the patients were treated with IVIG after 7 days of illness. Platelet (PLT) counts and ANXA3 levels concurrently exhibited significant increases 7 days after onset. Furthermore, ANXA3 levels were positively correlated with lymphocyte and PLT counts in the KD and KD-CAL groups. ANXA3 may be involved in the pathogenesis of KD and CALs.

## Introduction

Kawasaki disease (KD), also known as mucocutaneous lymph node syndrome, is classified as acute systemic vasculitis, and approximately 80% of affected patients are children under 5 years old (1, 2). Thrombocytosis has been reported in KD patients at 7 days of illness (3, 4). Coronary arterial lesions (CALs) are a major complication of KD (5). Platelets play an important role in injuring the vessel wall by affecting leukocyte functions and recruitment (6, 7). Although a standard regimen with intravenous immunoglobulin (IVIG) and aspirin is used, 15%–25% of patients still have CALs, including coronary arterial aneurysm (CAA), stenosis, myocardial infarction, ischemia, and even sudden death. Furthermore, KD has now become the main cause of acquired heart disease in children in developed countries (3, 8–10). Nonetheless, the pathogenesis of this disease remains largely unclear, and it is well known that an unknown stimulus induces the immune-mediated inflammatory cascade within the innate and adaptive immune systems of genetically susceptible children (11, 12). Due to the lack of a specific diagnostic test, the diagnosis of KD relies on clinical criteria. IVIG, acetylsalicylic acid (ASA), corticosteroids, infliximab, and etanercept are used in the treatment of KD in the acute phase to reduce inflammation and arterial damage. IVIG can prevent the development of CAA; however, there is still a 1% risk of giant aneurysm and a 5% risk of CAA (13, 14). It is necessary to find potential biomarkers for the early diagnosis and timely treatment of KD.

Annexins constitute a group of Ca^2+^-dependent soluble and hydrophilic proteins that reversibly bind to negatively charged phospholipid head groups (15). Studies have shown that annexins are involved in many cellular functions, such as proliferation, differentiation, apoptosis, and signal transduction, and play a key role in the regulation of the inflammatory response and blood anticoagulation properties (16). Annexin A3 (ANXA3), also known as 35-alpha calcimedin, lipocortin III or placental anticoagulant protein III, is a member of the annexin family (17). Although the physiological functions of ANXA3 are still not entirely clear, an increasing number of studies have suggested that ANXA3 not only regulates inflammation, migration and differentiation of cells but also promotes angiogenesis, stimulating neutrophil aggregation (18–21). Furthermore, previous studies reported that ANXA3 is involved in tumor invasion, intracranial aneurysm, the immunopathology of sepsis, and cardiovascular disease (22–25). Whether ANXA3 participates in regulating abnormal inflammation and the immunologic response in KD and CALs has not been reported thus far.

## Methods

### Subjects and data collection

Participants were recruited from the Children's Hospital of Chongqing Medical University, Chongqing, China. All of the KD cases strictly met the criteria proposed by the Scientific Committee of the Japanese Circulation Society (26, 27). Healthy children were recruited from physical examinations. There were 109 KD patients (76 males and 33 females; average age, 3.334 ± 2.083 years old) in the KD group and 58 healthy children (38 males and 20 females; average age, 3.443 ± 2.363 years old) in the control group (HC).

The diameter and *z* value of coronary arteries were assessed in the acute phase of KD by echocardiography. According to the *z* value of the coronary artery, KD patients were divided into two groups: the KD with CALs group (KD-CALs, *z* value >2.0, *n* = 67) and the KD without CALs group (KD-NCALs, *z* value <2.0, *n* = 42).

All blood samples were obtained from KD patients during the first week of onset before treatment with anticoagulants and intravenous immunoglobulin (IVIG). Then, the samples were centrifuged at 3000 rpm for 10 min, and the serum was stored at −80°C.

All experiments complied with the ethics committee from Chongqing Medical University, and written informed consent for all participants was provided before the study by their parents or legal guardians.

### Measurement of serum ANXA3 and clinical parameters

Serum concentrations of ANXA3 were quantified with a human ANXA3 ELISA kit (Cusabio, Wuhan, China) according to the manufacturer's instructions. The intra-assay and interassay precision were CV% < 8% and CV% < 10%, respectively.

The serum concentrations of ANXA3 were tested in KD and HC groups. The important clinical data of all the subjects with KD, including the duration days with fever and the time point of IVIG administration, were recorded. General laboratory data of all the subjects in KD were examined, including white blood cell counts (WBC), platelet counts (PLT), neutrophil counts, lymphocyte counts, alanine transaminase (ALT) levels, aspartate transaminase (AST) levels, albumin (ALB) levels, procalcitonin (PCT) levels, C-reactive protein (CRP) levels, the erythrocyte sedimentation rate (ESR) levels, and blood coagulation parameters such as thrombin time (TT), prothrombin time (PT), activated partial thromboplastin time (APTT) and plasma fibrinogen (FIB) levels. Eighty-eight KD patients had creatine kinase isoenzyme (CK-MB) results, 70 KD patients had brain natriuretic peptide (BNP) results, and 48 KD patients had cytokine results, including the levels of IL-6, IL-10, TNF-α, and INF-γ. Moreover, ANXA3 were tested in 15 paired KD-CAL patients both before and after day 7 disease onset.

### Statistical analysis

All data in this study are described as the mean ± standard deviation (SED) or median and interquartile range (IQR). Statistical significance was defined as a *P* value (2-tailed) < 0.05. Student's t test was used to compare data from two cohorts that followed a normal distribution, while the Mann–Whitney *U* test was used to compare nonnormally distributed data. The Wilcoxon signed ranks test was used to test paired samples for which data failed to follow a normal distribution. Correlations between serum ANXA3 levels and various laboratory parameters were assessed *via* Spearman rank correlation analysis.

SPSS 23.0 was used to analyze all statistical results. GraphPad Prism 8.0 and Adobe Illustrator 2021 were used to create the figures.

## Results

### ANXA3 levels in the HC group and KD group

**Figure 1:**
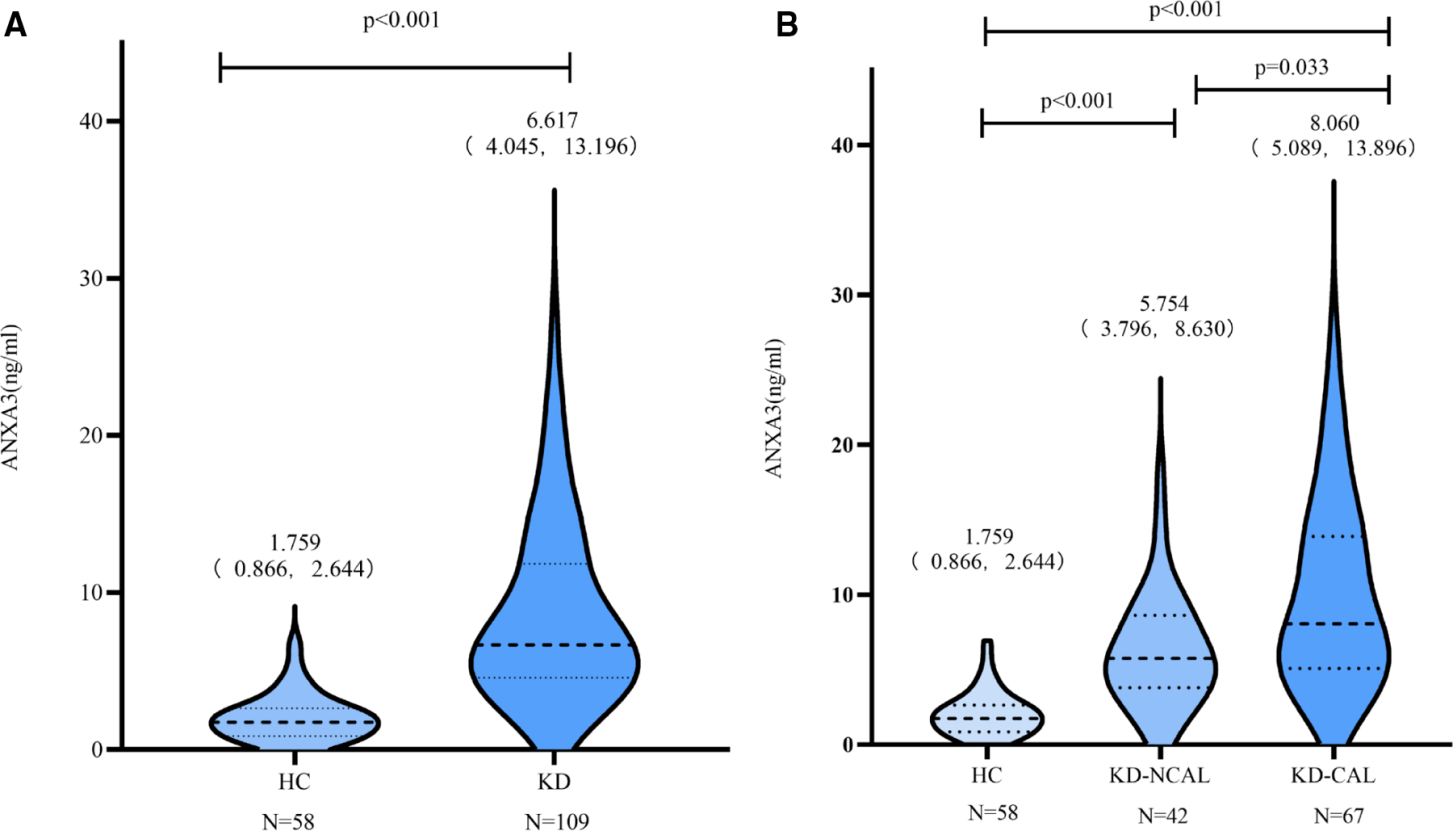
Serum levels of ANXA3 in the HC and KD groups A: ANXA3 levels in the HC and KD groups. B: ANXA3 levels in the HC, KD-NCALs and KD-CALs groups. Serum levels of ANXA3 was significant difference in the three groups (*P* < 0.001). The serum levels in HC group were significantly lower than that in KD-NCALs group and KD-CALs group (*P* < 0.001 both). The serum levels in KD-CALs group were significantly higher than that in KD-NCALs (*P* = 0.033). ANXA3: Annexin A3, HC: healthy children, KD: Kawasaki disease, KD-NCALs: Kawasaki disease non coronal arterial lesions, KD-CALs: Kawasaki disease with coronal arterial lesions.

There were no differences in age or sex between the KD and HC groups (*P* > 0.05). The levels of serum ANXA3 in the KD group [6.617 pg/ml (4.045 pg/ml, 13.196 pg/ml) *n* = 109] were significantly higher than those in the HC group [1.775 pg/ml (0.866 pg/ml, 2.657 pg/ml), *n* = 58] (*P* < 0.001). In addition, there were significant differences in the serum levels of ANXA3 among the KD-CAL group, KD-NCAL group and HC group (*P* < 0.001). The serum levels of ANXA3 in the KD-NCAL group [5.754 ng/ml (3.796 pg/ml, 8.630 pg/ml), *n* = 42] were higher than those in the HC group [1.775 pg/ml (0.866 pg/ml, 2.657 pg/ml), *n* = 58] (*P* < 0.001) but lower than those in the KD-CAL group [8.060 ng/ml (5.089 pg/ml, 13.896 pg/ml), *n* = 67] (*P* = 0.033) (Figure 1).

### Neutrophil cell counts in the HC and KD groups

**Figure 2:**
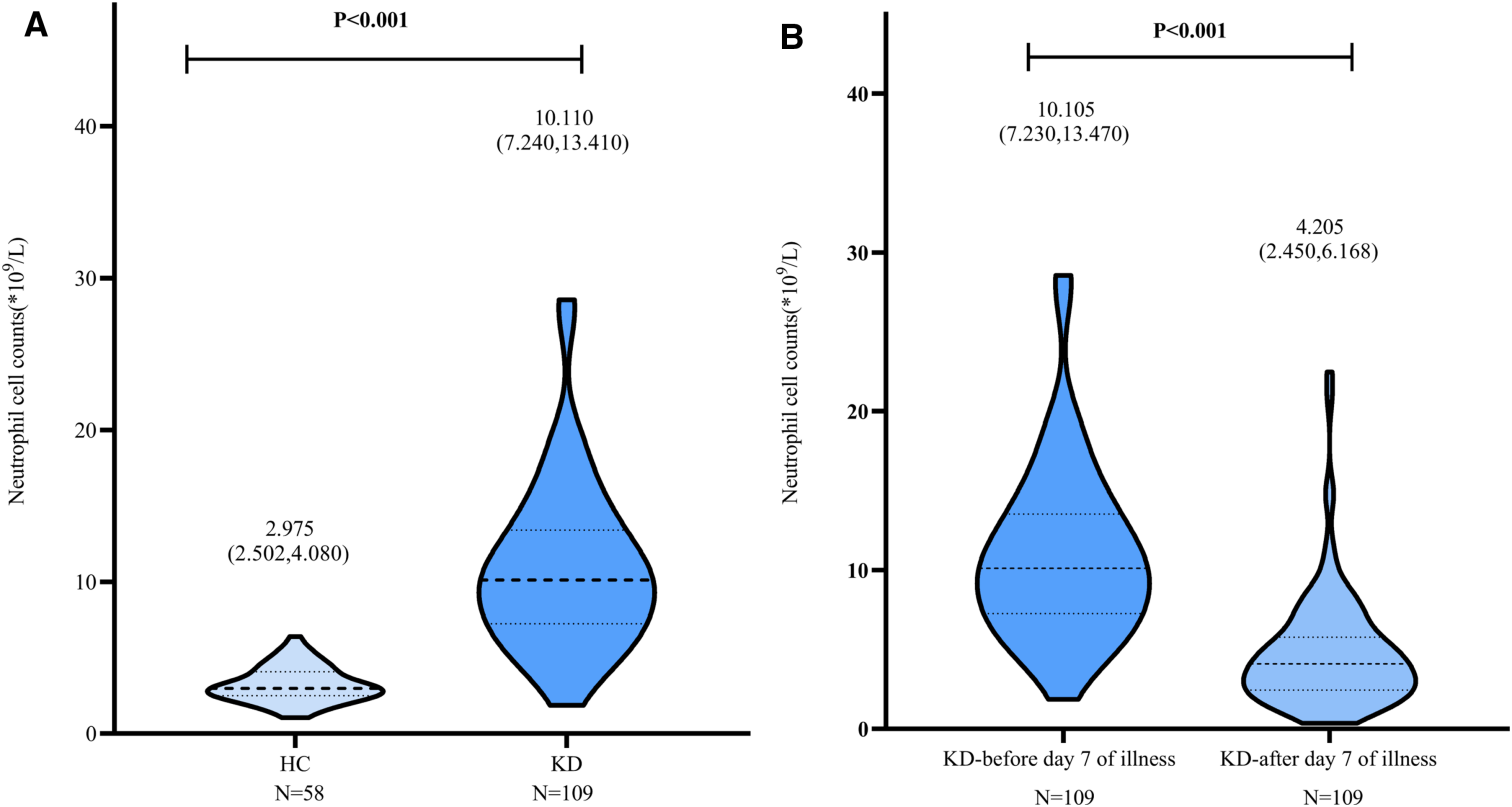
Neutrophil cell counts in HC group and KD group. A: The neutrophil cell counts in HC and KD groups. B: In KD group, the neutrophil cell counts decreased after day 7 of illness when treated with IVIG. IVIG: intravenous immunoglobulin.

The neutrophil cell counts were significantly elevated before day 7 of illness in the KD group [10.110 × 10^9^/L (7.240 × 10^9^/L, 13.410 × 10^9^/L), *n* = 109] in comparison with the HC group [2.975 × 10^9^/L (2.502 × 10^9^/L, 4.080 × 10^9^/L), *n* = 58] (*P* < 0.001). The neutrophil cell counts were markedly decreased when the patients were treated with IVIG after 7 days of illness [4.205 × 10^9^/L (2.450 × 10^9^/L, 6.168 × 10^9^/L), *n* = 109] (Figure 2).

### Correlation between ANXA3 levels and clinical parameters

There were distinctly positive correlations between ANXA3 and WBC (*r* = 0.227, *P* = 0.018) in the KD group and significant positive correlations with the time point of IVIG (day) (*r* = 0.242, *P* = 0.049) in the KD-CAL group. In addition, lymphocyte and PLT counts showed significant positive correlations with ANXA3 in the KD and KD-CAL groups (*r* = 0.226, *P* = 0.018; *r* = 0.276, *P* = 0.004 and *r* = 0.349, *P* = 0.004; *r* = 0.375, *P* = 0.002, respectively) (Table 1).

**Table 1:** Correlations between serum ANXA3 and clinical parameters in the KD, KD-CALs and KD-NCALs groups.

	*N* (KD,KD-NCALs,KD-CALs)	KD		KD-CALs		KD-NCALs	
		*r*	*P*	*r*	*P*	*r*	*P*
Time point of IVIG (day)	109,52,57	0.141	0.143	0.242	0.049*	−0.079	0.620
WBC (10^9^/L)	109,52,57	0.227	0.018*	0.143	0.247	0.291	0.061
Neutrophil (10^9^/L)	109,52,57	0.078	0.418	0.000	0.998	0.229	0.145
Lymphocyte (10^9^/L)	109,52,57	0.226	0.018*	0.349	0.004*	−0.242	0.123
PLT (10^9^/L)	109,52,57	0.276	0.004*	0.375	0.002*	0.132	0.406
ALT (U/L)	109,52,57	−0.052	0.595	−0.171	0.167	0.078	0.624
AST (U/L)	109,52,57	−0.070	0.469	−0.217	0.077	0.076	0.633
PCT (ng/ml)	109,52,57	−0.082	0.396	−0.220	0.073	0.111	0.483
APTT (s)	109,52,57	−0.016	0.866	−0.109	0.378	−0.039	0.807
Fib (g/L)	109,52,57	−0.038	0.698	−0.027	0.825	0.010	0.950
TT (s)	109,52,57	0.125	0.195	0.159	0.198	0.027	0.864
PT (s)	109,52,57	0.038	0.693	−0.115	0.356	0.266	0.089
ALB (g/L)	109,52,57	−0.042	0.668	−0.013	0.916	0.070	0.659
CRP (mg/L)	109,52,57	0.020	0.835	0.044	0.724	0.028	0.862
ESR (mm/h)	109,52,57	0.067	0.490	0.068	0.586	0.097	0.542
CK-MB (ug/L)	88,32,56	0.038	0.724	0.003	0.980	0.033	0.858
BNP (pg/ml)	70,25,45	−0.080	0.513	−0.008	0.956	−0.389	0.054
IL-6 (pg/ml)	48,19,29	0.040	0.785	-0.091	0.638	0.139	0.571
IL-10 (pg/ml)	48,19,29	0.008	0.956	-0.089	0.645	0.107	0.663
TNF-α (pg/ml)	48,19,29	-0.086	0.560	-0.094	0.629	-0.102	0.677
INF-γ (pg/ml)	48,19,29	-0.019	0.897	-0.067	0.729	0.010	0.969

Note: *P < 0.05.

### The relationship between serum ANXA3 and PLT counts in KD-CAL patients

**Figure 3:**
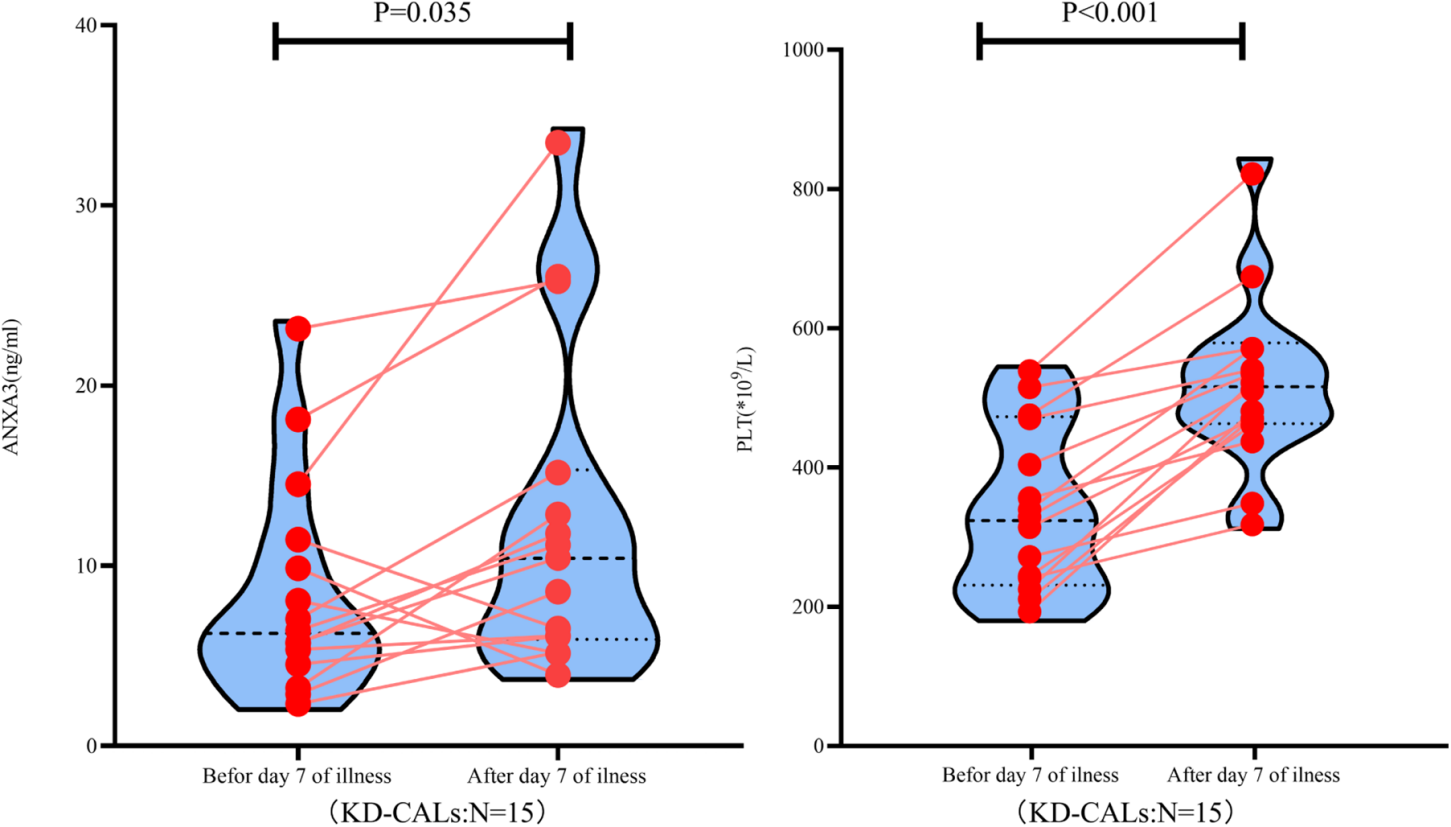
ANXA3 levels and PLT counts in paired KD patients (*n* = 15) both ANXA3 levels and PLT counts increased after day 7 of illness. ANXA3: Annexin A3, PLT: platelet counts, KD-CALs: Kawasaki disease with coronal arterial lesions.

Compared with the initial data obtained, the serum levels of ANXA3 and PLT counts showed a concurrent increase at day 7 after onset of illness in KD-CAL patients (Figure 3).

### The relationship between ANXA3 and other clinical and laboratory parameters in KD

There were significant differences in serum ANXA3, lymphocyte counts and serum albumin between the KD-NCAL and KD-CAL groups (*P* < 0.05) (Table 2).

**Table 2:** Laboratory data and serum ANXA3 levels in the KD-NCALs and KD-CALs group.

	*N* (KD-NCALs,KD-CALs)	KD-NCALs (IQR or SEM)	KD-CALs (IQR or SEM)	*P*-value
Time point of IVIG(day)	42,67	5.00(5.00,6.00)	6.00(5.00,7.00)	0.301
Duration of fever	42,67	6.50 (5.00,8.00)	7.00 (6.00,7.00)	0.636
ANXA3 (ng/ml)	42,67	5.75 (3.80,8.63)	8.06 (5.09,13.90)	0.002*
WBC (10^9^/L)	42,67	14.08 (10.53,16.15)	14.78 (11.53,18.69)	0.227
Neutrophil (10^9^/L)	42,67	10.09 (7.50,13.22)	10.15 (7.18,13.29)	0.950
Lymphocyte (10^9^/L)	42,67	2.38 (1.62,3.20)	3.10 (1.91,4.19)	0.042*
ALT (U/L)	42,67	39.50 (17.00,105.00)	53.00 (21.00,85.00)	0.540
AST (U/L)	42,67	29.00 (21.75,51.12)	31.00 (24.00,57.00)	0.317
PCT (ng/ml)	42,67	0.59 (0.27,1.95)	0.84 (0.25,2.97)	0.548
APTT (s)	42,67	29.60 (27.88,31.35)	30.70 (28.10,34.40)	0.065
FIB (g/L)	42,67	7.47 (5.67,8.10)	6.93 (6.04,7.65)	0.253
TT (s)	42,67	14.45 (14.28,14.73)	14.50 (14.10,15.10)	0.537
PT (s)	42,67	12.96 ± 1.53	12.89 ± 1.30	0.792
ALB (g/L)	42,67	36.18 ± 3.80	34.37 ± 4.56	0.034*
PLT (10^9^/L)	42,67	371.43 ± 101.50	365.67 ± 134.21	0.812
CRP (mg/L)	42,67	67.23 ± 32.93	63.87 ± 41.58	0.658
ESR (mm/h)	42,67	67.19 ± 23.47	67.95 ± 24.72	0.875
IL-6 (pg/ml)	19,29	328.72 (98.10,908.86)	453.51 (182.15,2208.52)	0.354
IL-10 (pg/ml)	19,29	13.18 (4.57,39.44)	14.22 (5.04,72.09)	0.643
TNF-α (pg/ml)	19,29	0.76 (0.01,2.59)	0.95 (0.12,4.97)	0.703
INF-γ (pg/ml)	19,29	0.92 (0.17,2.08)	2.28 (0.03,5.70)	0.642
CK-MB (ug/L)	32,56	0.76 (0.60,1.04)	52.86 (20.12,166.09)	0.775
BNP (pg/ml)	25,45	26.41 (11.37,94,73)	52.86 (20.12,166.09)	0.223

Note: *Compared with the KD-NCALs groups *P* < 0.05.

### The effect of ANXA3 in predicting KD and KD-CALs

**Figure 4:**
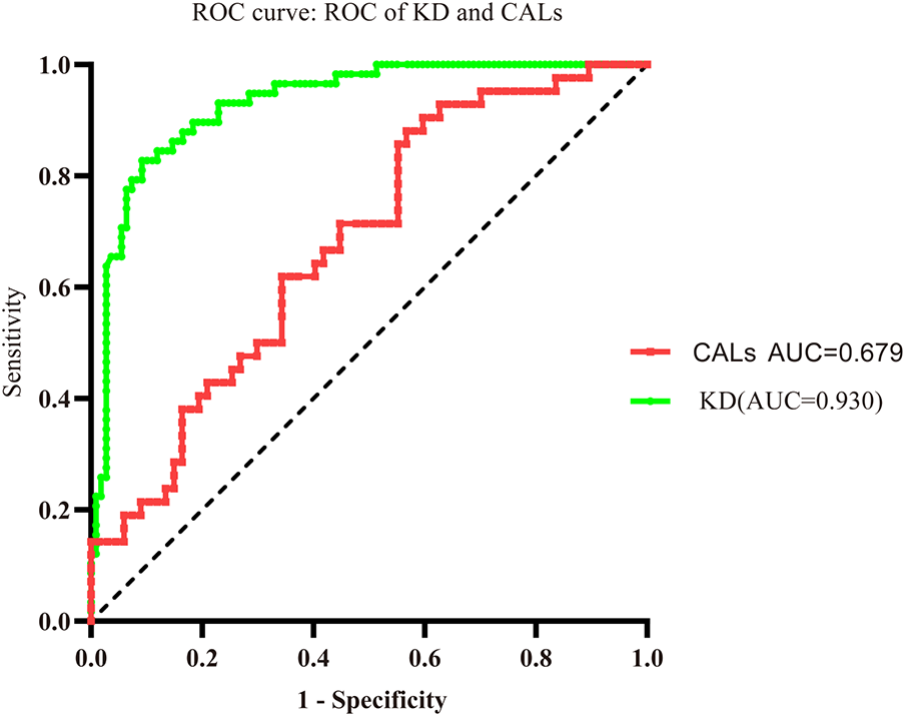
Evaluation of serum ANXA3 for diagnosis of patients with KD and KD-CALs. Receiver operating characteristic (ROC) curves of ANXA3 in KD and KD-CALs groups.

The AUC values of the KD group and KD-CAL group were 0.930 and 0.679, respectively (95% confidence intervals: 0.892 to 0.969 and 0.578 to 0.779, respectively; *P* < 0.001 and *P* = 0.002, respectively). The cutoff values were 3.033 pg/ml and 9.846 pg/ml, respectively (Figure 4).

## Discussion

KD as the main cause of acquired cardiovascular disease in children. It is presumably triggered by an infection-related abnormal inflammatory reaction in genetically susceptible children. The abnormal response of immune cells and vascular endothelial dysfunction are important causes of CALs in patients with KD (28). Finding a biomarker for the early diagnosis of KD has attracted much attention due to the lack of a “gold standard” for diagnosis. In our study, we examined the serum levels of ANXA3 in all subjects. The results showed that the serum levels of ANXA3 were increased in the KD group and were higher in the KD-CAL group than in the KD-NCAL group. Thus, we may predict that ANXA3 may be involved in the pathogenesis of KD and CALs.

First, studies showed that ANXA3 is expressed by neutrophils and monocytes, particularly in neutrophils (18, 29). ANXA3 consists of 323 amino acid residues and is encoded by a water-soluble protein-coding gene located on chromosome 4q13-q22 in the human genome. There are four conserved domains (I–IV) forming a circular structure. Each of the domains consists of five α-helices (helices A-E) and forms the C-terminal region of ANXA3. Two tryptophan residues are crucial for the interaction of calcium ions and phospholipid membranes in ANXA3 and are involved in various physiological events (30, 31). ANXA3 has been correlated with tumorigenesis, cell signal transduction, apoptosis, anticoagulation, and angiogenesis (21, 31, 32). Studies have examined the role of ANXA3 in immunity and inflammation and shown that ANXA3 promotes membrane-membrane contact in neutrophils, and furthermore, phagocytic vesicle membranes fuse with the membranes of neutrophil granules. Imaging data also demonstrated that in neutrophils, ANXA3 was redistributed to phagosomes (33, 34). In KD, neutrophils have been suggested to be among the first cells to invade the arterial wall and are the most important cells that participate in the first stage of arteritis (2, 35, 36). In our study, the serum levels of ANXA3 and neutrophil cell counts were increased in the acute phase of KD. ANXA3 is particularly abundant in neutrophils, accounting for ∼1% of all intracellular proteins. Increased serum levels of ANXA3 7 days before the onset of illness may be associated with neutrophil infiltration in KD. It can be speculated that ANXA3 and neutrophils may take part in the process of coronary arterial lesions in KD. Then, an increasing number of studies have confirmed the function of platelets not only in the regulation of hemostasis but also in immunity and inflammation (37, 38). Proteomic analysis has indicated that platelets can secrete more than 300 proteins to regulate inflammation and the immune response. Moreover, Joaquim, H.P.G et al. (39) reported that ANXA3 was expressed in platelets in schizophrenia patients. Additionally, Suades, R et al. (40) showed that Annexin family members were present in extracellular vesicles released from platelets. Laboratory tests confirmed that thrombocytosis occurs 7 days after illness and is a typical feature of the subacute phase of KD (2, 3). Platelets can act as the first responders in the recognition of pathogens. They rapidly generate host-defense peptides and then recruit and boost leukocytes to promote T cell-B cell crosstalk, which is necessary in adaptive immunity (41–43). In addition, platelets play an important role in injuring the vessel wall (4, 44). This study also demonstrated that there was a marked correlation between ANXA3 levels and PLTs, not only in the levels but also in the timing of increase during disease progression among KD patients. In addition, ANXA3 had a positive correlation with PLTs in the KD group and KD-CAL group. This suggests that ANXA3 and PLTs may be involved together in the pathogenesis of KD.

In this study, the higher level of serum ANXA3 was accompanied by an increase in the neutrophil cell count, and when KD patients were treated with IVIG, the neutrophil cell count decreased. However, serum ANXA3 remained at a higher level, so the higher levels of serum ANXA3 may come from PLTs. In addition, Meng H et al. demonstrated that the silencing of ANXA3 promotes the repair and healing of myocardial tissue through PI3K/Akt signaling pathway activation in rats with acute myocardial infarction (45). The ROC curves of ANXA3 showed that ANXA3 predicted KD and KD-CALs. Accordingly, we postulated that ANXA3 may be the intermediary agent between the neutrophils and PLTs involved in the pathogenesis of KD and further in the development of CALs. The pathogenesis of ANXA3 involvement in neutrophils and PLTs, which injure vascular endothelial cells in KD and CALs needs further study.

In conclusion, this study first verified that the serum concentration of ANXA3 was markedly higher in the KD group than in the HC group and higher in the KD-CAL group than in the KD-NCAL group. There was a positive relationship between ANXA3 and WBCs, lymphocytes and PLTs. The timing of the ANXA3 concentration increase coincides with neutrophil and PLT counts. Accordingly, we postulate that ANXA3 may be an intermediary agent between neutrophils and PLTs in the pathogenesis of KD and further in the development of CALs in different stages of KD. Therefore, ANXA3 may be a potential target in the prevention of KD and KD-CALs.

This study had limitations. One is due to the small number of KD samples, further studies are need to demonstrated the signaling pathway of ANXA3, neutrophil and PLTs that is involved in the pathogenesis of KD and CALs. The other is all the patients were enrolled from in a single center, multicenter experimental evidence were needed in the further.

## Data Availability

The original contributions presented in the study are included in the article/Supplementary Material, further inquiries can be directed to the corresponding authors.
